# Field Emission of Multi-Walled Carbon Nanotubes from Pt-Assisted Chemical Vapor Deposition

**DOI:** 10.3390/nano12030575

**Published:** 2022-02-08

**Authors:** Hongbin Tang, Ruizi Liu, Weijun Huang, Wei Zhu, Weijin Qian, Changkun Dong

**Affiliations:** Wenzhou Key Lab of Micro-nano Optoelectronic Devices, Wenzhou University, Wenzhou 325035, China; tanghb152@163.com (H.T.); liurz@163.com (R.L.); 18857757816@163.com (W.H.); 184511084138@stu.wzu.edu.cn (W.Z.); weijinqian@wzu.edu.cn (W.Q.)

**Keywords:** multi-walled carbon nanotubes, metal substrate, platinum, assistant growth, chemical vapor deposition, field emission

## Abstract

Multi-walled carbon nanotubes (MWNTs) were grown directly on a metal substrate with the assistance of Pt using a chemical vapor deposition method. In addition, the growth mechanism of Pt-assisted catalytic CNT was discussed. MWNTs were characterized by SEM, TEM, AFM, Raman, and EDS, and the field emission (FE) properties were investigated, comparing with the direct grown MWNTs. The results showed that CNTs could not been synthesized by Pt particles alone under the experimental condition, but Pt may accelerate the decomposition of the carbon source gas, i.e., assisting MWNT growth with other catalysts. The Pt-assisted MWNTs were longer with larger diameters of around 80 nm and possessed better structural qualities with very few catalyst particles inside. Improved field emission properties were demonstrated for the Pt-assisted MWNTs with lower turn-on fields (for 0.01 mA·cm^−2^ current density) of 2.0 V·μm^−1^ and threshold field (for 10 mA·cm^−2^ current density) of 3.5 V·μm^−1^, as well as better stability under a long-term test of 80 h (started at 3.0 mA for the Pt-assisted emitter and 3.25 mA for the direct grown emitter). This work demonstrated a promising approach to develop high performance CNT field emitters for device applications.

## 1. Introduction

Due to unique structural, chemical, and physical properties, carbon nanotubes (CNTs) have been applied and investigated in various areas [1,2,3,4]. With the advantages of large aspect ratio, high electrical conductivity, and good thermal stability, CNTs present excellent field emission properties for different types of vacuum electronic devices [5,6,7]. However, there are still many issues for practical applications, including emission uniformity and emission stabilities in high pressure ambiences. The improvement of CNT film production with emission enhancement is crucial to promote the device’s developments.

Among various carbon nanotube synthesis techniques, chemical vapor deposition (CVD) is widely employed for simple processing, in situ growth on various substrates, and low cost. In most CVD synthesis, carbon nanotubes are grown from the catalyst films, e.g., Fe, Co, and Ni, and deposited on the substrates [8,9]. Bronikowski investigated the effects of multiple factors on the synthesis of carbon nanotube bundle arrays with Fe catalyst on Si substrates [10]. Terrado et al. prepared uniform multi-walled carbon nanotubes by spraying precipitated Co catalyst particles on quartz substrates and discussed the effects of catalyst pretreatment and growth temperature on CNT diameters [11]. The direct growth of CNTs on metallic substrates containing catalyst elements exhibited significant advantages, including the enhancement of adhesion and reduction in contact resistance between CNTs and the substrates, resulting in improvements in field emission properties. Direct CNT growth demonstrates great potentials for field emission device applications [5,12,13,14]. Beyond typical single element catalyst (e.g., Fe, Co, Ni, etc.), multi-element catalysts, such as Ni alloys, have been found to present prominent catalytic effects for CNT synthesis [5,15,16,17]. CNT growth mechanisms from multi-catalyst systems are mostly ambiguous. Kiang et al. suggested that synergistic effects of large bi-metal catalyst, such as Co and Bi, result in the expansion of CNT diameters [18]. Teng et al. synthesized CNTs from the Co-Cr-Pt-O catalytic system and attributed simply the synergistic effect for growth [19]. Researchers also reported that tungsten, molybdenum, and sulfur could be used as the auxiliary catalyst to improve catalyst activity and increase the yield of carbon nanotubes [20,21]. Bimetallic catalyst combination (Ni + Co) resulted in 10–100 times higher growth rate than those of nickel or cobalt alone in the arc discharge synthesis of single-wall CNTs [22]. Sridhar et al. synthesized MWNTs on Inconel alloy substrates by water-assisted chemical vapor deposition with good field emission properties from high local field enhancement factors [23].

In carbon nanotube synthesis, Pt is rarely used as the catalyst. Iijima et al. successfully prepared single-walled carbon nanotubes (SWNTs) by ethanol with Pt as the catalyst, where SWNTs with diameters smaller than 1 nm were synthesized under low pressure (1 × 10^−4^ Pa) and low temperature (400 °C), showing that Pt has high catalytic activity [24,25]. Synthesis of MWNT films on the catalytic substrates, mainly transition metal-based alloy materials, showed advantages of process simplicity and substrate flexibility [26,27,28]. In this work, we proposed a novel direct MWNT growth method on Pt coated Ni alloy substrates. Structural and field emission properties are characterized by comparing with the MWNT emitters by the direct growth method. Pt-assisted MWNT emitters exhibited better structural quality with significant improvements on field emission performances.

## 2. Materials and Methods

Ni alloy substrates (Ni:Cr:Fe:Co = 57.5:15.5:6:1.5) of 0.2 mm in thickness were employed for the investigation. For direct growth, the substrate was anodized in the oxalic acid solution and ultrasonically cleaned with ethanol and ultrapurified water. For Pt-assisted growth, the substrate was coated with Pt by magnetron sputtering under a power of 40 W. Pt film was 35 nm thick measure by the film thickness detector of the sputtering system (VTC-16-SM, Kejing, Hefei, China). The sputtering chamber was evacuated to about 2 Pa at first, and then Ar gas was introduced to increase the pressure to 9 Pa before sputtering the high-purity Pt target (purity: 99.99%). After sputtering, the substrate was placed in the CVD system, which was evacuated to about 4 Pa by a mechanical pump and heated to 500 °C with purging Ar gas as a carrier gas. After reaching 750 °C, C_2_H_2_ was introduced to increase the pressure to 1.0 × 10^3^ Pa for 10 min to grow the MWNTs. In the contrast, the MWNT emitters on the alloy substrate was also directly synthesized by the above CVD process without Pt deposition. Structural and field emission properties were comparatively investigated.

The structural and elemental properties of MWNTs were characterized by scanning electron microscopy (SEM, JSM-7100F, JEOL, Tokyo, Japan), transmission electron microscopy (TEM, JEM-2100, JEOL, Tokyo, Japan), Atomic Force Microscope (AFM, Nanowizard 3, JPK, Berlin, Germany), and energy-dispersive X-ray analysis (EDS). The contact resistance of the MWNT film relative to the substrate was tested by the dual electrodynamic four-probe method by the Probe Station (PEH-4, EverBeing, Taiwan, China) and Keithley 2440 multimeter (Tektronix, State of Oregon, USA). Field emission performances were investigated in a high vacuum turbo system with the base pressure in 10^−7^ Pa level after baking the system at 250℃ for 12 h. In the diode field emission setup, the CNT emission film area was 12 mm^2^, and the cathode and anode were spaced 300 μm apart. The Keithley 248 power supply was employed to provide the emission potential.

## 3. Results

### 3.1. Structural Characterization of Carbon Nanotubes

**Figure 1:**
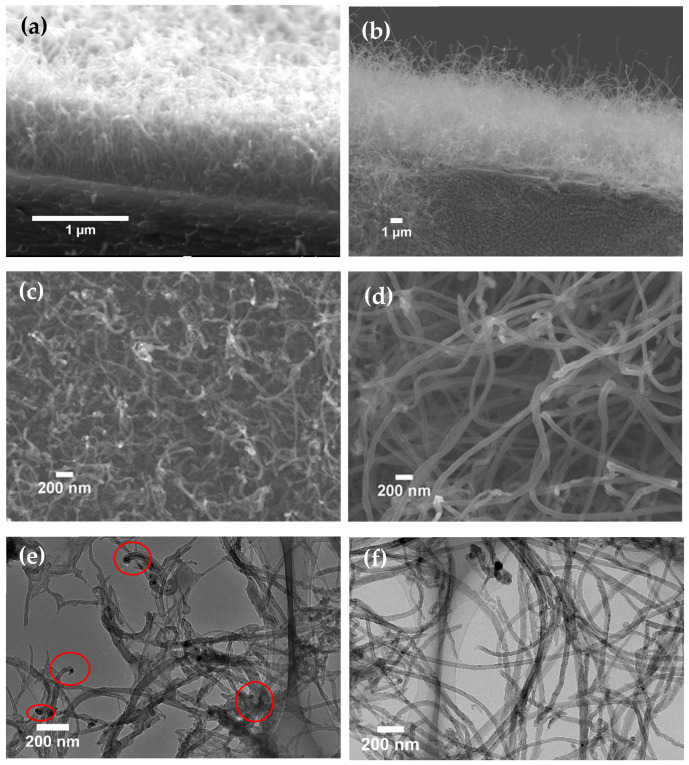
SEM and TEM images of two types of MWNTs. (**a**,**c**,**e**) direct growth. (**b**,**d**,**f**) Pt-assisted growth.

**Figure 2:**
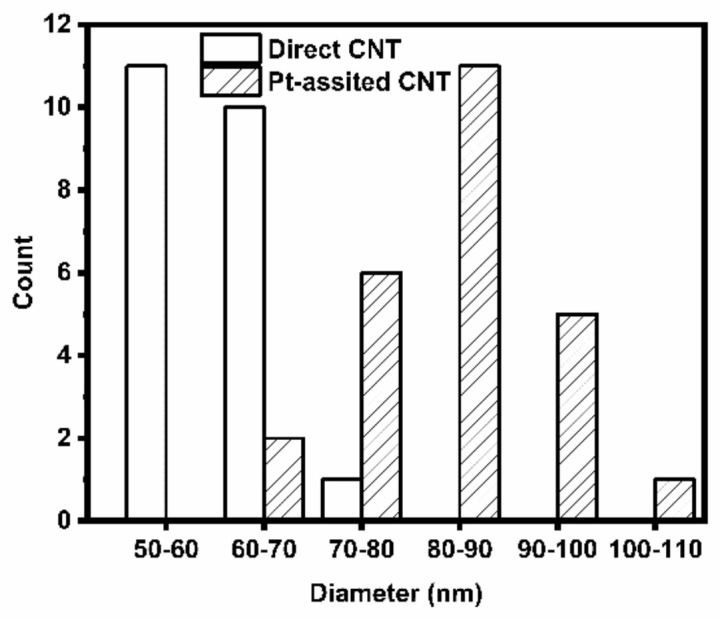
Diameter distributions of two types of MWNTs.

Both Pt-assisted and the direct grown MWNTs were randomly oriented and exhibit good structural homogeneities, as shown in Figure 1. However, the addition of Pt elements affected CNT growth significantly. The Pt-assisted MWNTs could be 10 μm long with larger tube diameters in the 70~90 nm range, while the direct grown MWNTs were shorter and denser with lengths of ~1 μm and diameters in the 50~70 nm range (Figure 2). Random orientation and good homogeneity are favorable for reducing emission hot spots and the edge effect from local excessive Joule heating under high emission to burn the long CNT away [29,30]. According to high magnification SEM (Figure 1c,d) and TEM (Figure 1e,f) images, the Pt-assisted MWNTs show less amorphous carbon to outer walls with smoother surfaces. There were fewer catalyst particles inside Pt-assisted MWNTs, which would help to improve electronic and thermal conductivities. In contrast, more catalyst particles were detected along direct growth MWNTs, especially inside the caps.

**Figure 3:**
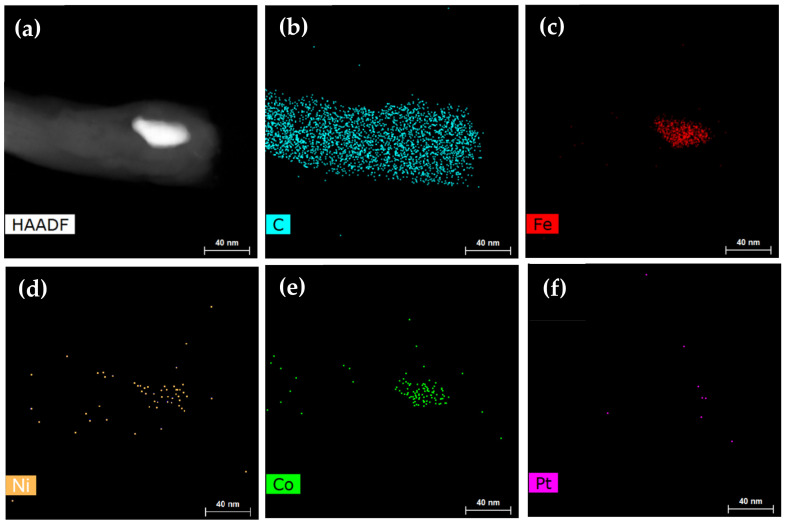
TEM-EDS mapping of Pt-assisted MWNT. (**a**) MWNT with catalyst particle inside the top. (**b**–**f**) Element distributions for C, Fe, Ni, Co, and Pt.

What is rarer is that there still exist catalyst particles inside Pt-assisted MWNTs. Thus, EDS mapping was applied to identify catalyst components. As shown in Figure 3, beside the carbon element, the catalyst particle inside the tube top consisted of Fe, Co, and Ni elements, which were from the metal substrate, but the Pt element was not identified, with the exception of scattered noise signals.

**Figure 4:**
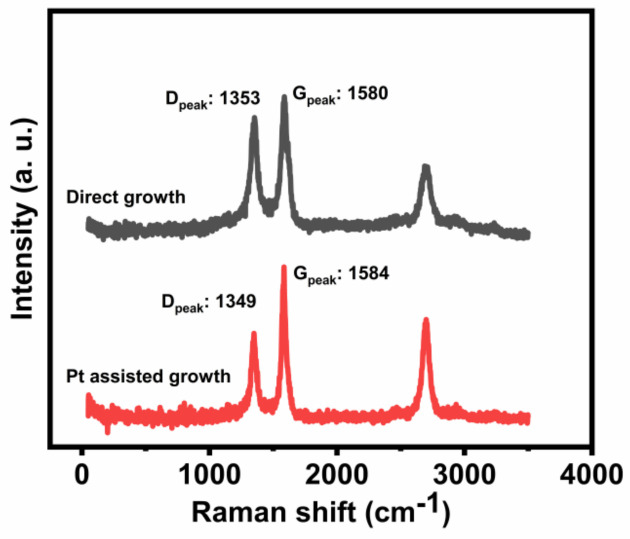
Raman spectra of two types of MWNTs.

Raman characteristic performances were investigated for two types of MWNTs, as shown in Figure 4. The D peak at 1360 cm^−1^ and G peak at 1584 cm^−1^ correspond to vibration modes of defect structures and crystal graphite, respectively. The D peak to G peak ratio is always used to evaluate CNT crystallinity, and the smaller the I_D_/I_G_, the better the structural quality. The I_D_/I_G_ ratio of direct grown MWNTs was 0.73, which is better than MWNTs on the Inconel alloy substrate [23,31], while the I_D_/I_G_ of Pt-assisted MWNTs could reach 0.56, indicating improvements on structural perfection.

**Figure 5:**
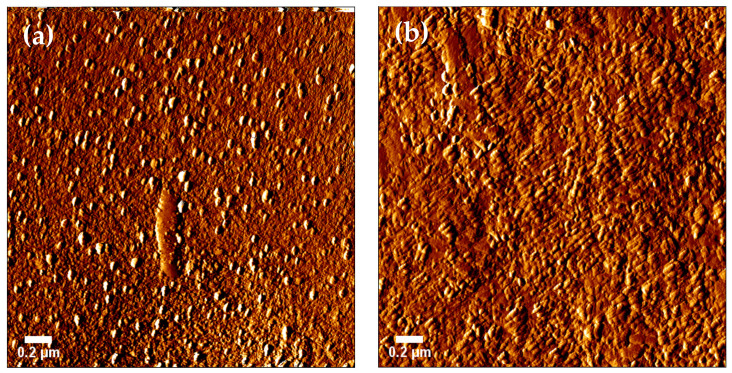
AFM images of the surface morphologies of two types of substrates before the growth. (**a**) Direct growth substrate. (**b**) Pt-coated substrate.

**Figure 6:**
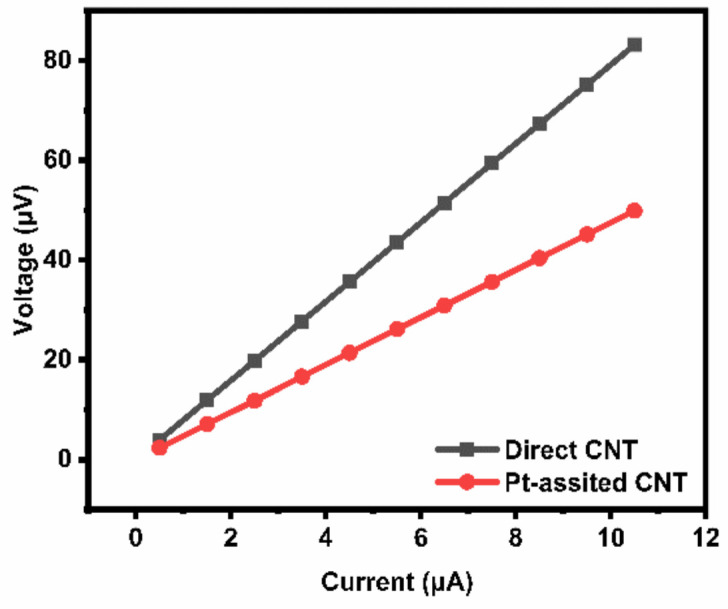
Contact resistance of two types of MWNTs.

The surface morphologies of two types of substrates before the growth were analyzed. Figure 5 shows AFM analysis of the substrates right before MWNT growth, i.e., after heating at 750 °C in Ar for 10 min. The catalyst particles of about 60 nm, which were in the same dimension of the MWNT diameters, are believed to serve as the growth catalysts, distributed uniformly on the substrate surface for direct growth (Figure 5a). For the Pt-coated substrate, the surface was in tight bonded form with narrow belt shape bulges (Figure 5b). The EDS result showed that the Pt element covered the entire substrate surface (Figure S1). For comparison, we failed to grow CNTs for either sputtering Pt films on Si substrates or depositing Pt films of more than 50 nm on alloy substrates, implying that the single Pt element could not catalyze the CNT synthesis alone based on our growth system. The contact resistance properties of the MWNT films relative to the substrates were characterized, as shown in Figure 6. The contact resistances from the slope are 4.68 Ω and 7.86 Ω, respectively, for Pt-assisted and direct growth MWNT films.

Based on the fact that Pt-assisted MWNTs exhibited very few catalyst particles inside, it may suggest a bottom growth mechanism. Pt film could play a crucial role in holding catalyst particles with the substrate, which is also supported by the fact that there were no Pt elements identified inside the tube from the element mapping above. The better electrical contact and field emission properties below also indicate stronger adhesions between Pt-assisted MWNTs relative to the substrate, supporting the bottom growth mechanism. According to cross-sectional SEM images of MWNTs, it was found that Pt-assisted MWNTs are about ten times longer compared with direct growth tubes under the same growth conditions. Therefore, Pt nanoparticles may accelerate the decompositions of acetylene molecules to grow longer and larger diameter MWNTs with better structural quality.

### 3.2. Field Emission Performance Test

**Figure 7:**
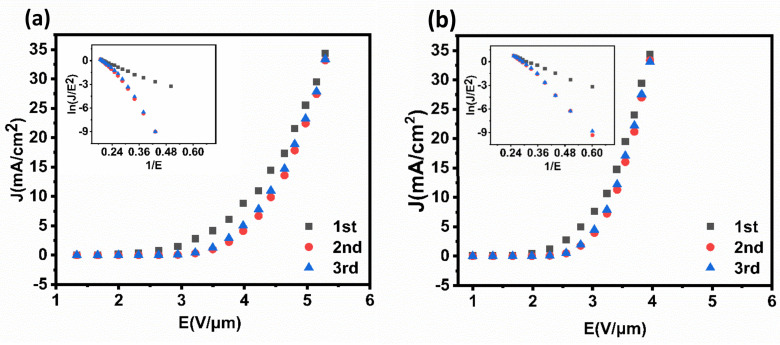
Field emission J−E properties for two types of MWNT emitters with insets of F−N curves. (**a**) Direct growth MWNTs. (**b**) Pt-assisted growth.

Field emission performances of current density vs. electric field are illustrated in Figure 7 for both emitters (the field emission uniformity of the Pt-assisted MWNT film is shown in Figure S2), with insets of corresponding to Fowler–Nordheim (F-N) curves. Pt-assisted MWNTs showed better field emission characteristics than direct growth MWNT emitters. In three test cycles, the first cycle deviated from the second and third cycles, which are attributed to the desorptions of gas adsorbents [6,32]. Then, the second and third tests have good reproducibility. The turn-on field (for 0.01 mA·cm^−2^ current density) and the threshold field (for 10 mA·cm^−2^ current density) were 2.0 V·μm^−1^ and 3.5 V·μm^−1^, respectively, for the Pt-assisted emitter and improved significantly comparing with 2.7 V·μm^−1^ and 4.65 V·μm^−1^ for the direct grown emitter. According to the following F-N equation:

(1) $$\beta =-6.83\times 10^{3}\times \varphi^{\frac{3}{2}}/\mathrm{slope}$$

where β is the field enhancement factor, φ is the work function (~4.8 eV for MWNT), and the slope is taken from the fitted F-N curve from the third and second test cycles. Thus, β values for the Pt-assisted and direct grown MWNTs are 2486 and 1942, respectively. It is clear that reductions of turn-on and threshold fields of the Pt-assisted MWNTs are attributed mainly to the increase in β.

**Figure 8:**
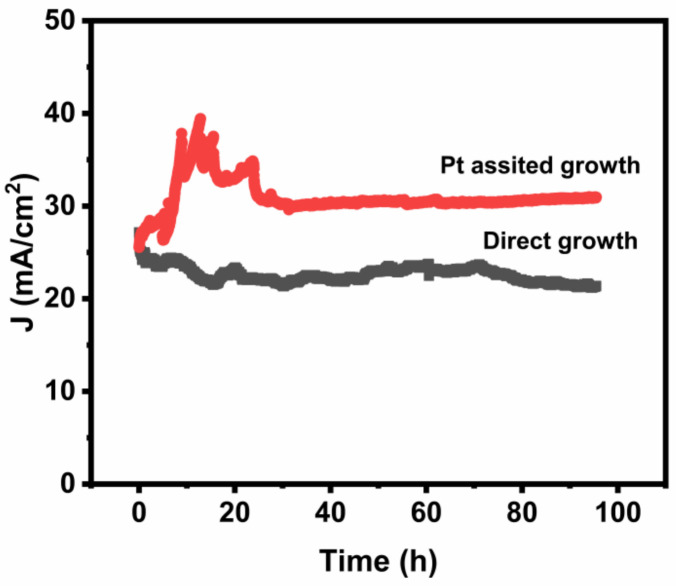
Emission stability tests of two types MWNT emitters.

The field emission stabilities of both types of emitters were tested in the high vacuum system, as shown in Figure 8. The emission currents started at 3.0 mA (25 mA·cm^−2^) for the Pt-assisted emitter and 3.25 mA (27 mA·cm^−2^) for the direct grown emitter. For the Pt-assisted emitter, the current fluctuated between 3.2 mA and 4.8 mA in the first 20 h and then stabilized at 3.6 mA for 80 h. For the direct grown emitter, the emission current dropped to 2.6 mA in 15 h, then reached stable emission around 2.8 mA with a fluctuation rate of ±1.25%. The initial current increase for the Pt-assisted emitters could be attributed to the CNT stretching effect by electrostatic force from the electric field [33,34], especially for curved long tubes, resulting in an increase in the field enhancement effect and a decrease in CNT–anode distance. It was suggested that the high emission current may cause uniform melting in the CNT-catalyst–substrate interface, which improves electrical conductivity and mechanical strength at the interface [20]. Such effects may be more obvious for the Pt-assisted emitters, which could have better adhesions with the substrate for the bottom growth model, helping the initial current increase. After such a stretching period, poorly bonded MWNTs may be pulled off from the substrate. Meanwhile, emission hot spots could be burned away by over current Joule heating, resulting in the current decay. After such aging process, emission reached quite stable levels with a fluctuation rate of less than 1%. While for the direct grown emitters, the tube was shorter; thus, the stretching effect was weak. Therefore, during the initial aging period, burning away hot spots and low crystallinity tubes may play key roles, resulting in emission decline.

## 4. Conclusions

MWNT field emitters were synthesized on a Pt-coated Ni alloy substrate by thermal CVD, and the structural and field emission properties were investigated in comparison with the direct grown MWNTs. The Pt-assisted MWNTs were longer with larger diameters of around 80 nm and possessed better structural qualities with very few catalyst particles inside. The Pt-assisted MWNTs exhibited improved field emission characteristics with the turn-on field of 2.0 V·μm^−1^ and the threshold field of 3.5 V·μm^−1^. MWNTs presented excellent emission stability under the current density of ~30 mA·cm^−2^ with a current fluctuation of less than 1%. This work presented a new approach to synthesize CNT field emitters for device applications.

## Data Availability

The data presented in this study are available on request from the corresponding author. The data are not publicly aviable due to privacy restrictions.

## Supplementary Materials

The following are available online at https://www.mdpi.com/article/10.3390/nano12030575/s1, Figure S1: EDS mapping of Pt coated substrate after heating, Figure S2: Field emission uniformity of Pt assisted MWNT film with emission area of 12 mm^2^.
