# Supplementary material for: Field Emission of Multi-Walled Carbon Nanotubes from Pt-Assisted Chemical Vapor Deposition
Source: Nanomaterials (Basel). 2022 Feb 8;12(3):575. doi: 10.3390/nano12030575 (PMC8838496; doi:10.3390/nano12030575)
Supplement: Supplementary file 1 [file nanomaterials-12-00575-s001.zip › nanomaterials-1560232-supplementary.pdf]

Additional Information

Field Emission of Multi-Walled Carbon Nanotubes from Pt Assisted Chemical Vapor Deposition

Hongbin Tang , Ruizi Liu , Weijun Huang, Wei Zhu, Weijin Qian, and Changkun Dong*

Wenzhou Key Lab of Micro-nano Optoelectronic Devices, Wenzhou University, Wenzhou, Zhejiang 325035, People's Republic of China; Tanghb152@163.com (H.T.); liurz@163.com (R.L.); 18857757816@163.com (W.H.); 184511084138@stu.wzu.edu.cn (W.Z.); weijinqian@wzu.edu.cn (W.Q.); *Correspondence: dck@wzu.edu.cn; Tel: +86-577-86689067 (C.D.)

Index

Figure S1. EDS mapping of Pt coated substrate after heating

Figure S2. Field emission uniformity of Pt assisted MWNT film with emission area of 12 mm^2

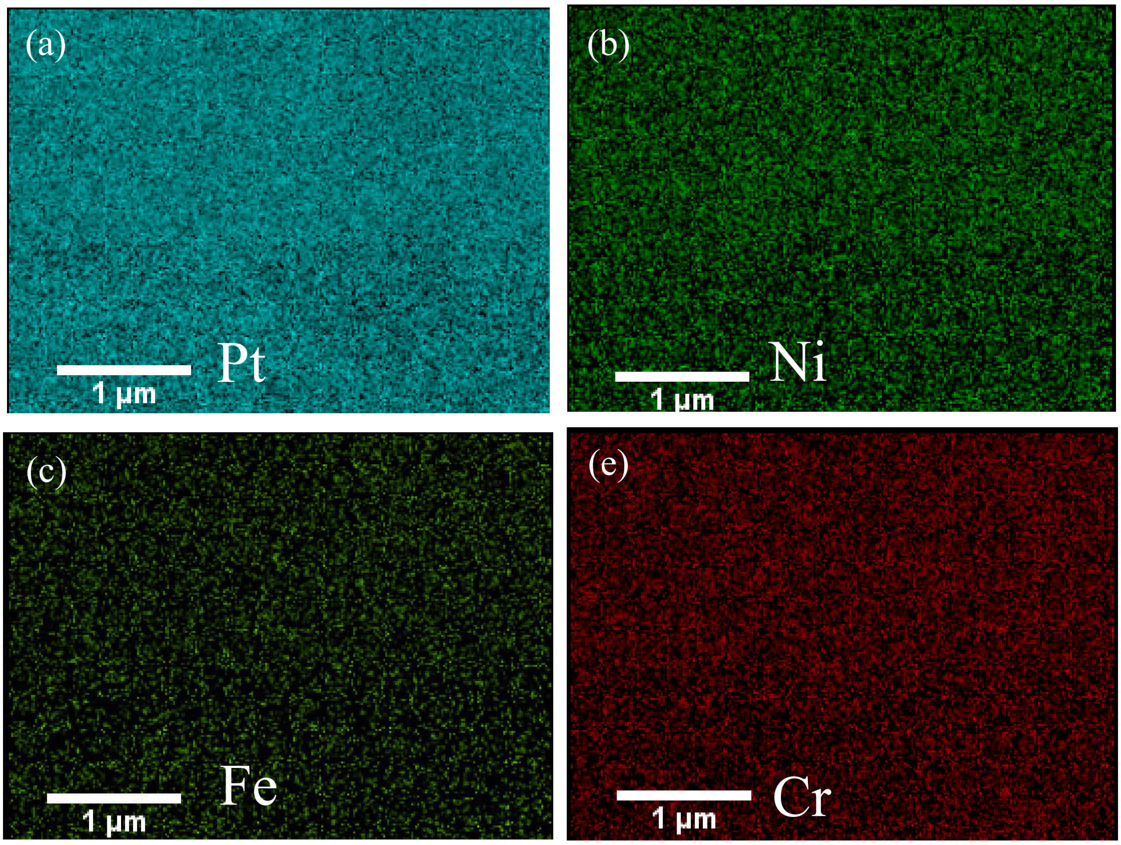

Figure S1. EDS mapping of Pt coated substrate after heating

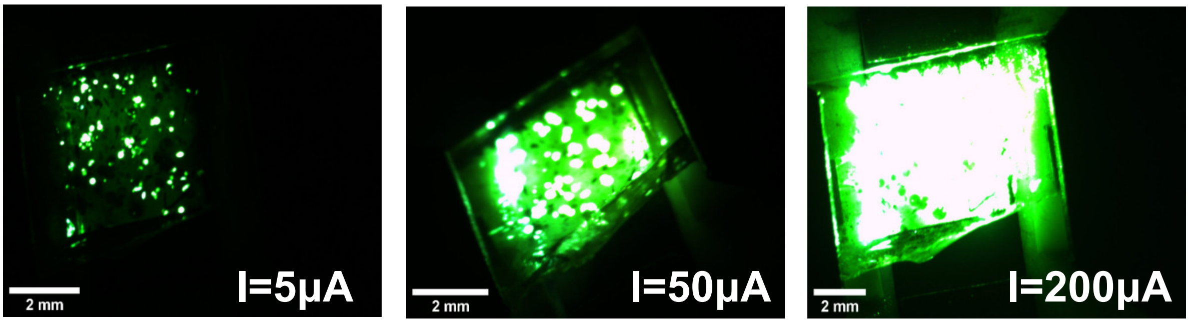

Figure S2. Field emission uniformity of Pt assisted MWNT film with emission area of 12 mm^2
